# Transcranial direct current stimulation over left dorsolateral prefrontal cortex facilitates auditory-motor integration for vocal pitch regulation

**DOI:** 10.3389/fnins.2023.1208581

**Published:** 2023-06-30

**Authors:** Yichen Chang, Danhua Peng, Yan Zhao, Xi Chen, Jingting Li, Xiuqin Wu, Peng Liu, Hanjun Liu

**Affiliations:** ^1^Department of Rehabilitation Medicine, The First Affiliated Hospital, Sun Yat-sen University, Guangzhou, China; ^2^Guangdong Provincial Key Laboratory of Brain Function and Disease, Zhongshan School of Medicine, Sun Yat-sen University, Guangzhou, China

**Keywords:** auditory feedback, speech motor control, left dorsolateral prefrontal cortex, transcranial direct current stimulation, top–down modulation

## Abstract

**Background:**

A growing body of literature has implicated the left dorsolateral prefrontal cortex (DLPFC) in the online monitoring of vocal production through auditory feedback. Specifically, disruption of or damage to the left DLPFC leads to exaggerated compensatory vocal responses to altered auditory feedback. It is conceivable that enhancing the cortical excitability of the left DLPFC may produce inhibitory influences on vocal feedback control by reducing vocal compensations.

**Methods:**

We used anodal transcranial direct current stimulation (a-tDCS) to modulate cortical excitability of the left DLPFC and examined its effects on auditory-motor integration for vocal pitch regulation. Seventeen healthy young adults vocalized vowel sounds while hearing their voice pseudo-randomly pitch-shifted by ±50 or ±200 cents, either during (online) or after (offline) receiving active or sham a-tDCS over the left DLPFC.

**Results:**

Active a-tDCS over the left DLPFC led to significantly smaller peak magnitudes and shorter peak times of vocal compensations for pitch perturbations than sham stimulation. In addition, this effect was consistent regardless of the timing of a-tDCS (online or offline stimulation) and the size and direction of the pitch perturbation.

**Conclusion:**

These findings provide the first causal evidence that a-tDCS over the left DLPFC can facilitate auditory-motor integration for compensatory adjustment to errors in vocal output. Reduced and accelerated vocal compensations caused by a-tDCS over left DLPFC support the hypothesis of a top–down neural mechanism that exerts inhibitory control over vocal motor behavior through auditory feedback.

## Introduction

Auditory feedback is an essential part of speech motor control, providing sensory information that allows spakers to monitor and adjust their vocal out to produce their intended speech goals (Smotherman, 2007). This control process is known as auditory-motor integration for speech production, typically manifested as compensatory adjustment of vocal motor behavior in response to any mismatches between expected and actual auditory feedback in voice fundamental frequency (*f*_o_), intensity, or formant frequency (*F*_1_) (Burnett et al., 1998; Houde and Jordan, 1998; Bauer et al., 2006). Using various neuromaging techqnies including functional magnetic resonance imaging (fMRI), magnetoencephalography (MEG), electroencephalography (ECoG), and event-realted potential (ERP), a growing body of literature has revealed a complex, widely distributed network located in the frontal, parietal and temporal regions as well as subcortical areas (Houde and Nagarajan, 2011; Behroozmand et al., 2015; Guenther and Hickok, 2015; Behroozmand et al., 2018). These regions are thought to detect auditory feedback errors and generate corrective motor commands to control speech production. The precise roles of these brain regions in auditory-vocal integration, however, remain far from clear.

The prefrontal cortex, particularly the inferior frontal gyrus (IFG), has been considered to be an essential region that supports vocal feedback control. For instance, the directions into velocity of articulators (DIVA) model proposes that the left IFG contains a speech sound map that initiates the feedback and feedforward control of speech production (Golfinopoulos et al., 2010). Similarly, the dual stream model posits that the left IFG serves as a core component of a dorsal stream responsible for mapping acoustic speech signals onto articulatory representations (Hickok and Poeppel, 2007). In line with these models, empirical evidence has identified activation of the IFG and its connectivity with temporal and parietal regions in producing vocal adjustments to auditory feedback errors (Flagmeier et al., 2014; Behroozmand et al., 2015; Kort et al., 2016).

In contrast, little attention has been paid to the dorsolateral prefrontal cortex (DLPFC) in the context of auditory-vocal integration. The DLPFC encompasses a large brain region characterized by considerable structural heterogeneity, spanning over Brodmann areas 9, 8a, 8b, and the dorsal part of 46 (Glasser et al., 2016). This structural complexity of the DLPFC positions it as a key brain region for involvement in a variety of cognitive functions, including working memory (Edin et al., 2009), attentional control (Brosnan and Wiegand, 2017), and executive functions (Mansouri et al., 2009). Notably, these cognitive functions have been implicated in auditory-vocal integration. For example, focused attention led to enhanced vocal compensations for pitch perturbations and/or ERP P2 response while divided attention reduced them (Tumber et al., 2014; Liu et al., 2015). As well, engagement of working memory during vocal pitch regulation led to increased vocal compensations and ERP N1 amplitudes but decreased ERP P2 amplitudes in response to pitch perturbations (Guo et al., 2017). Moreover, patients with Alzheimer’s disease (AD) exhibited abnormally enhanced magnitudes and reduced durations of vocal compensations for pitch perturbations that were significantly correlated with their executive and memory dysfunctions (Ranasinghe et al., 2017). These findings suggest that the DLPFC may contribute to auditory-motor integration for vocal production in a top-down manner.

A few neuroimaging studies have provided direct evidence supporting the involvement of the DLPFC in vocal feedback control. For example, Zarate and Zatorre (2008) reported activation of the left DLPFC in non-singers who were instructed to ignore or compensate for perceived pitch perturbations during singing. Ranasinghe et al. (2019) found that patients with AD exhibited significantly larger vocal compensations for pitch perturbations and lower left DLPFC activity than healthy controls, with lower left DLPFC activity predicting larger vocal compensations across both groups. One possible explanation for these abnormalities in vocal feedback control associated with AD is the impairment of prefrontal mediated inhibition (Ranasinghe et al., 2017). More recently, Liu et al. (2020) found that inhibiting the left DLPFC with continuous theta burst stimulation (cTBS), a non-invasive brain stimulation (NIBS) technique that induces inhibitory effects on cortical excitability (Huang et al., 2005), led to enhanced vocal compensations and reduced ERP P2 amplitudes in response to pitch perturbations. This finding establishes a causal link between the left DLPFC and auditory-motor integration for vocal production.

Building upon the essential role of the left DLPFC in suppressing reflex-like or inappropriate behavioral responses (Loftus et al., 2015; Angius et al., 2019) and modulating auditory processing (Knight et al., 1989; Mitchell et al., 2005), Liu et al. (2020) proposed that the left DLPFC may exert top-down control over the interaction between auditory and motor representations of vocal sounds to inhibit compensatory adjustment for feedback perturbations, thereby preventing vocal motor control from being excessively influenced by auditory feedback. This top-down mechiansm mediated by the left DLPFC generates an inhibitory influence on auditory feedback control of vocal production. Dysfunction of this mechanism may account for abnormally ehnaced vocal compensations for feedback errors when the left DLPFC was impaired (Ranasinghe et al., 2017, 2019) or inhibited (Liu et al., 2020). Conversely, it is reasonable that enhancing activity in the left DLPFC may produce inhibitory influences on vocal feedback control. This hypothesis is supported by the findings of Guo et al. (2017), showing that extensive training of working memory that is primarily subserved by the DLPFC decreased vocal compensations and increased ERP P2 amplitudes in response to pitch perturbations. However, direct causal evidence in support of this hypothesis is still lacking.

Therefore, the present study aimed to fill this gap by using transcranial direction current stimulation (tDCS), another NIBS technique that modulates cortical excitability by delivering an electric current to the scalp through electrodes (Nitsche and Paulus, 2000), to increase left DLPFC activity and investigate whether it can produce inhibitory effects on vocal feedback control. Generally, anodal tDCS (a-tDCS) increases cortical excitability whereas cathodal tDCS (c-tDCS) decreases it (Stagg and Nitsche, 2011). Previous studies have shown that a-tDCS over the left DLPFC increases its cortical excitability, as indicated by increased EEG power or fMRI activation in the frontal regions (Keeser et al., 2011; Zaehle et al., 2011). A large body of literature has shown that tDCS over brain regions can influence cognitive or motor functions in healthy and clinical populations (Mancuso et al., 2016; Lefaucheur et al., 2017; Manor et al., 2021; Tedla et al., 2023). Recently, tDCS has been used to investigate the neural mechanisms of auditory-motor integration for vocal production from a causal perspective. For example, Behroozmand et al. (2020) found that a-tDCS and c-tDCS over the left ventral motor cortex led to decreased vocal compensations for downward pitch perturbations compared to sham stimulation, with stronger effects associated with c-tDCS. In contrast, a-tDCS over the left sensorimotor cortex led to increased adaptive responses to *F*_1_ perturbations during speech production (Scott et al., 2020). In addition, vocal compensations for pitch perturbations became significantly larger when a-tDCS was applied over the right cerebellum relative to sham stimulation (Peng et al., 2021).

The frequency-altered feedback (FAF) paradigm was used to assess the effects of a-tDCS on vocal feedback control in the present study, during which participants produced sustained vocalizations while hearing their voice pitch-shifted unexpectedly. Participants received either active or sham a-tDCS over the left DLPFC during (online stimulation) or before (offline stimulation) the FAF task. The timing of tDCS was manipulated in the present study, as previous studies have reported inconsistent results regarding the optimal timing of tDCS for congitive performance or motor learning (Stagg et al., 2011; Mancuso et al., 2016; Buchwald et al., 2019). Additionally, Peng et al. (2021) found both online and offline a-tDCS over the right cerebellum led to enhacemed vocal compensations for pitch perturbations. Buidling upon the findings of abnormally enhanced vocal compensations for pitch perturbations when activity of the left DLPFC was disrupted by inhibitory c-TBS (Liu et al., 2020) or impaired due to AD (Ranasinghe et al., 2019), we hypothesized that increasing cortical exicitability of the left DLPFC with a-tDCS would result in reduced vocal compensations comapred to sham stimulation. Consistent with this hypothesis, our results showed smaller vocal responses to pitch perturbations following a-tDCS over the left DLPFC, providing further evidence for its invovlment in top-down inhibitory control over vocal motor behavior.

## Materials and methods

### Subjects

Twenty students [11 females and 9 males; age (mean ± SD): 21.8 ± 2.2 years] from Sun Yat-sen University were enrolled in this study. All participants met the following criteria: right-handed; native Mandarin speaker; no hearing or speech impairment; no history of neurological diseases; no use of neuropsychiatric drugs; no implanted medical devices such as pacemakers; not pregnant; and no claustrophobia. Three participants were excluded from the statistical analysis because they did not produce sustained vocalizations in a steady manner as required, resulting in the failure of extracting reliable voice *f*_o_ contours from their voice signals. Therefore, their data had to be excluded from the present study, and the final data pool contained the data from 17 participants [9 females and 8 males; age (mean ± SD): 21.4 ± 2.1 years]. All participants provided written informed consent and the research protocol was approved by the Institutional Review Board of The First Affiliated Hospital of Sun Yat-sen University in accordance with the Code of Ethics of the World Medical Association (Declaration of Helsinki).

#### Transcranial direct current stimulation

Direct current stimulation was administered by a battery-driven, constant current-stimulator (model EM8060, E&M Medical Tech., China). The present study consisted of two stimulation conditions: anodal stimulation and sham stimulation. In both conditions, a 6 cm × 4 cm electrode was placed over the F3 position on the scalp according to the 10–20 International System of EEG electrode placement to target the left DLPFC (Herwig et al., 2003) and a reference electrode with the same size was placed on the right deltoid muscle. During the anodal stimulation, a constant current of 1 mA was administered for 20 min with a 30 s ramp up/down phase at the beginning and end (Nitsche and Paulus, 2000). During the sham stimulation, the current was turned off after 30 s when it reached 1 mA.

#### Experimental procedure

This was a randomized, crossover study with four sessions: online active a-tDCS, online sham a-tDCS, offline active a-tDCS, and offline sham a-tDCS. Each session was conducted at least 48 h apart to eliminate the possible carry-over effects. Participants received active or sham a-tDCS over left DLPFC (i.e., online stimulation) while vocalizing the /u/ sound for 6 s following a blue light cue on the computer screen. During each vocalization, participants heard their voice pitch-shifted upwards or downwards by 50 or 200 cents (200 ms duration) in a pseudo-randomized manner. The direction and size of pitch perturbations were varied because previous studies have shown their effects on vocal compensation behavior (Chen et al., 2007; Liu et al., 2011; Scheerer et al., 2013). Additionally, Behroozmand et al. (2020) found that tDCS over left ventral motor cortex reduced vocal compensations only for downward pitch perturbations. A number of five pitch perturbations were pseudo-randomly presented within each vocalization, with the first pitch perturbation occurring 1,000–1,500 ms after the utterance onset and the subsequent ones at 700–900 ms inter-stimulus intervals. To avoid vocal fatigue, participants were required to take a break of 6 s prior to initiating the next vocalization. For the offline stimulation sessions, participants received active or sham a-tDCS over left DLPFC for 20 min before performing the vocalization task with the same parameters as the online stimulation sessions. Within each stimulation session, participants produced 40 consecutive vocalizations that led to a total of 200 trials, with 50 trials for each of the four perturbations (+50, −50, +200, and −200 cents). Notably, sponge electrodes were placed on the scalp during the online active a-tDCS session but were removed from the scalp in the offline active a-tDCS session. To control for the potential confounding effects of subject expectation or motivation between the online and offline stimulation conditions, two different sham conditions were implemented in this study: sponge electrodes were kept on the scalp during the online sham session, but removed during the offline sham session.

#### Data acquisition

All participants performed the FAF-based vocal production experiment in a sound-attenuated booth. A dynamic microphone (DM2200, Takstar Inc.) was used to pick up the voice signals, which were amplified by a MOTU Ultralite Mk3 Firewire audio interface to 10 dB SPL above the participants’ voice level to reduce the masking effects of the air-and bone-conducted feedback. The voice signals were then pitch-shifted by an Eventide Eclipse Harmonizer controlled by a custom-developed MIDI software program (Max/MSP, v5.0 by Cycling 74). This program also generated the transistor-transistor logic (TTL) control pulse that marked the onset of the pitch shift and the visual cues that instructed the participants to start and stop the vocalizations. The pitch-shifted voice signals were delivered back to the participants through insert earphones (ER-1, Etymotic Research Inc.) after amplification by an ICON NeoAmp headphone amplifier. A PowerLab A/D converter (ML880, AD Instruments) digitized the original and feedback voice signals and TTL control pulses at 10 kHz, and LabChart software (v7.0, AD Instruments) recorded them on an iMAC computer.

#### Data analyses

As previously described in Peng et al. (2021), an IGOR PRO software program (v6.0, Wavemetrics Inc.) was developed to measure the magnitude and latency of vocal compensations for pitch perturbations across the conditions. In brief, the voice *f*_o_ contours in Hertz was extracted from the voice signals using Praat software (Boersma, 2001) and converted into cents scale according to the following formula: cents = 100 × (12 × log_2_(*f*_o_/reference)) [reference = 195.997 Hz (G3 note)].Then they were segmented into epochs from 100 ms before to 700 ms after the onset of the pitch perturbation. All individual trials were visually inspected to reject bad trials that were contaminated by vocal interruptions or signal processing errors. Artifact-free trials were averaged to generate an overall vocal response to pitch perturbations for each condition, followed by a base-correction procedure that subtracts the mean *f*_o_ value in the baseline period (−100 ms to 0) from the *f*_o_ value after the perturbation onset. The peak *f*_o_ value in cents and the peak time in ms were considered as the magnitude and latency of a vocal response when the voice *f*_o_ contours reached their minimum or maximum value.

#### Statistics analyses

Repeated-measures analysis of variances (RM-ANOVAs) were used to analyze the values of vocal responses to pitch perturbations in SPSS (v.20.0). To investigate the online or offline effects of a-tDCS over left DLPFC, the magnitudes and latencies of vocal responses were subjected to three-way RM-ANOVAs with three factors: stimulation condition (a-tDCS vs. sham), perturbation magnitude (50 vs. 200 cents), and perturbation direction (upwards vs. downwards). In addition, four-way RM-ANOVAs were conducted to examine where the effects of a-tDCS varied as a function of the stimulation timing was delivered, including factors of stimulation timing (online vs. offline), stimulation condition, perturbation size and perturbation direction. Any significant higher-order interactions among these factors led to subsidiary RM-ANOVAs, and Bonferroni correction was used for *post hoc* multiple comparisons. Probability values for multiple degrees of freedom were corrected using Greenhouse–Geisser in the case of violation of the assumption of Mauchly’ test of Sphericity. Partial η^2^ ( $\eta_{p}^{2}$ ) was calculated as an index of effect size to quantify the proportion of variance. *p*-values <0.05 were considered significant.

## Results

### Effects of online a-tDCS over left DLPFC

**Figure 1:**
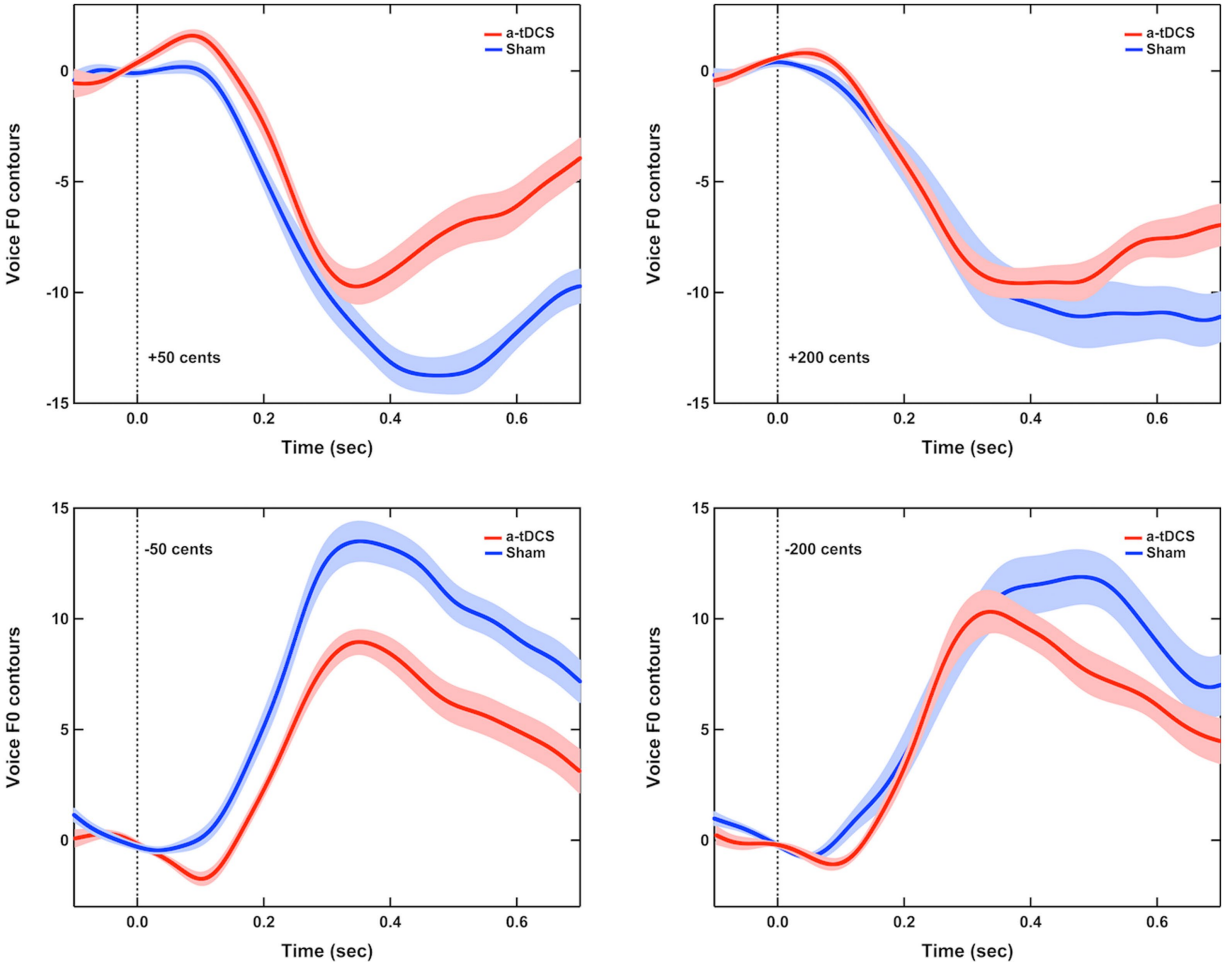
Grand-averaged voice *f*_o_ responses to pitch perturbations of ±50 cents **(left panel)** and ±200 cents **(right panel)** when active (red solid lines) or sham (blue solid lines) a-tDCS over the left DLPFC was applied during the FAF task. Highlighted areas represent the standard errors of the mean vocal responses, while the vertical dash lines indicate the onset of pitch perturbations.

**Figure 2:**
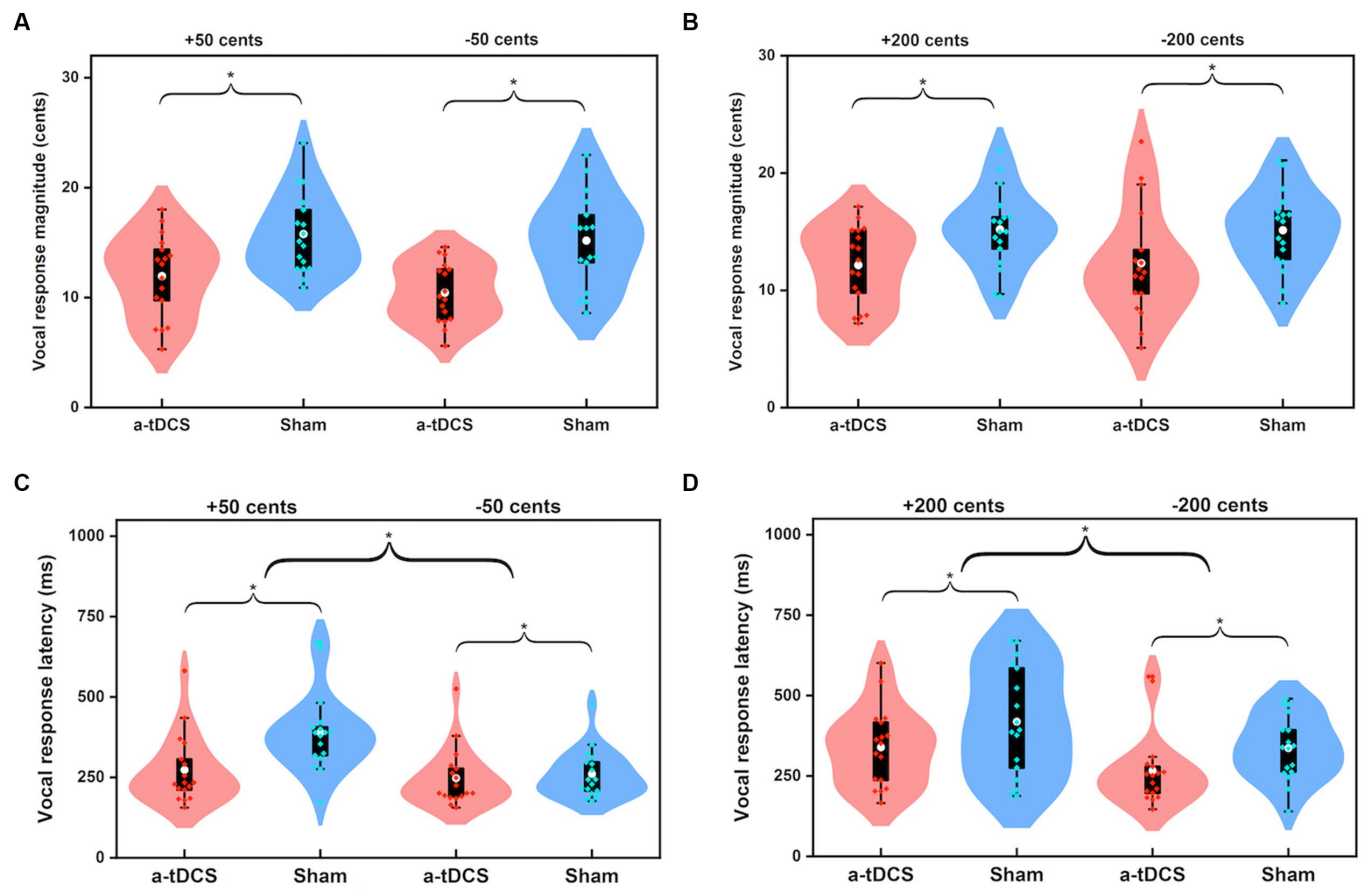
Violin plots illustrating the magnitudes **(A,B)** and latencies **(C,D)** of vocal responses to pitch perturbations of ±50 cents and ±200 cents when active (red) and sham (blue) a-tDCS over the left DLPFC was applied during the FAF task. The shape of the violin shows the kernel density estimate of the data. The white dots and box plots represent the medians and ranges from first to third quartiles of the data sets. The red and blue dots represent the individual vocal responses for active and sham a-tDCS over the left DLPFC. The asterisks indicate significant differences across the conditions.

Figure 1 shows the grand-averaged voice *f*_o_ responses to perburations of ±50 and ±200 cents during active or sham a-tDCS over the left DLPFC. A three-way RM-ANOVA conducted on the peak magnitudes of vocal responses revealed a significant main effect of stimulation condition [*F*(1, 16) = 24.473, *p* < 0.001, $\eta_{p}^{2}$ =0.605], indicating that online a-tDCS over the left DLPFC elicited smaller vocal responses than sham stimulation (see Figures 2A,B). However, the magnitudes of vocal responses did not vary as a function of perturbation size [*F*(1, 19) = 0.451, *p* = 0.512] or direction [*F*(1, 16) = 1.080, *p* = 0.314]. In addition, there were no significant interactions among the three factors (*p* > 0.3).

For the peak latencies of vocal responses, there was a significant main effect of stimulation condition [*F*(1, 16) = 14.142, *p* = 0.002, $\eta_{p}^{2}$ =0.469], indicating that a shorter time was required to reach the peak magnitude of vocal response for online a-tDCS over the left DLPFC relative to sham stimulation (see Figures 2C,D). Also, upward perturbations elicited significantly longer peak latencies of vocal responses than downward perturbations [*F*(1, 16) = 16.595, *p* = 0.001, $\eta_{p}^{2}$ =0.509], and 200 cents perturbations elicited significantly longer peak latencies of vocal responses than 50 cents perturbations [*F*(1, 16) = 9.265, *p* = 0.008, $\eta_{p}^{2}$ =0.367]. The interactions among the three factors were not significant (*p* > 0.1).

### Effects of offline a-tDCS over left DLPFC

**Figure 3:**
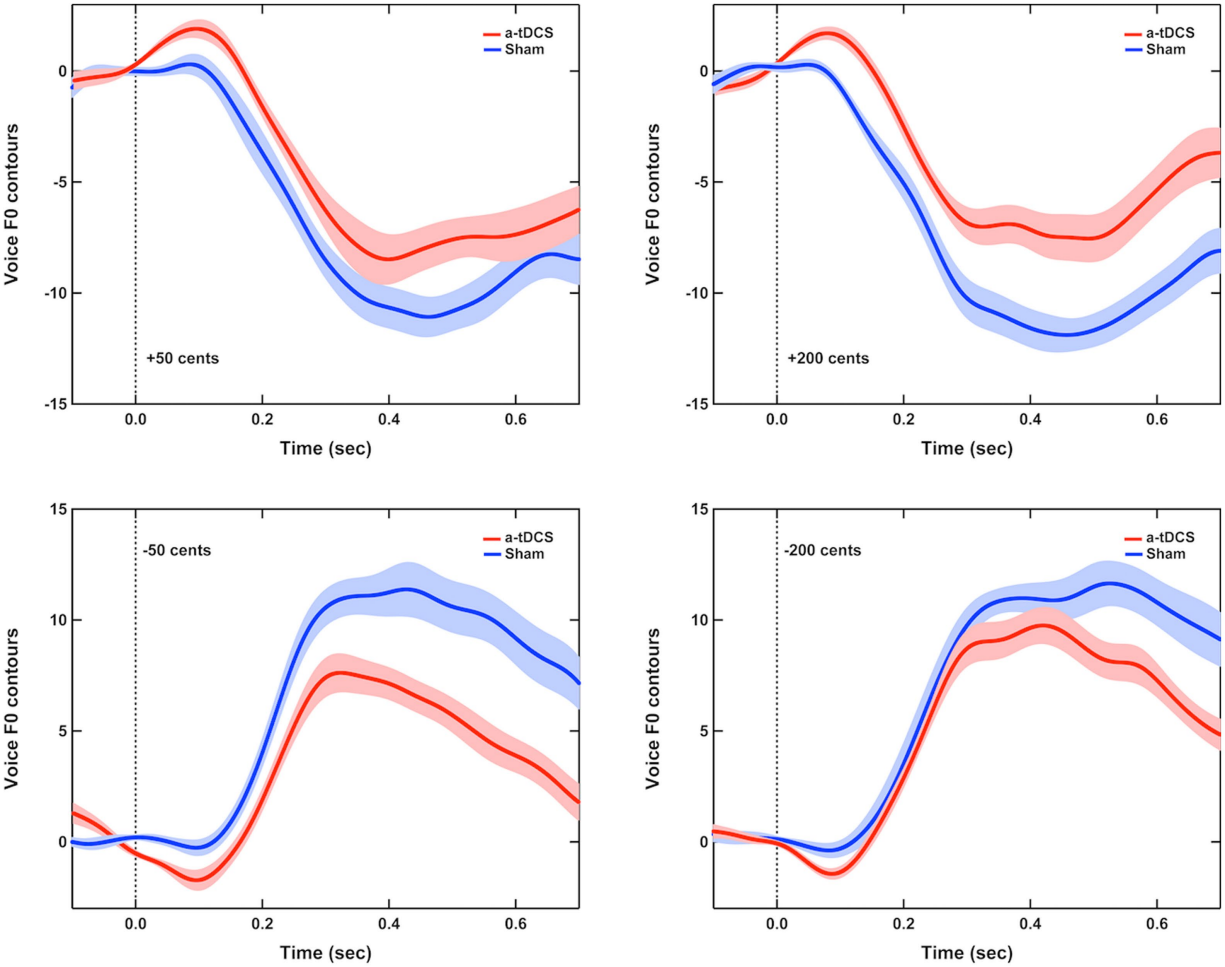
Grand-averaged voice *f*_o_ contours in response to pitch perturbations of ±50 cents **(left panel)** and ±200 cents **(right panel)** when active (red solid lines) or sham (blue solid lines) a-tDCS was applied over the left DLPFC prior to the FAF task. Highlighted areas represent the standard errors of the mean vocal responses, while the vertical dash lines indicate the onset of pitch perturbations.

**Figure 4:**
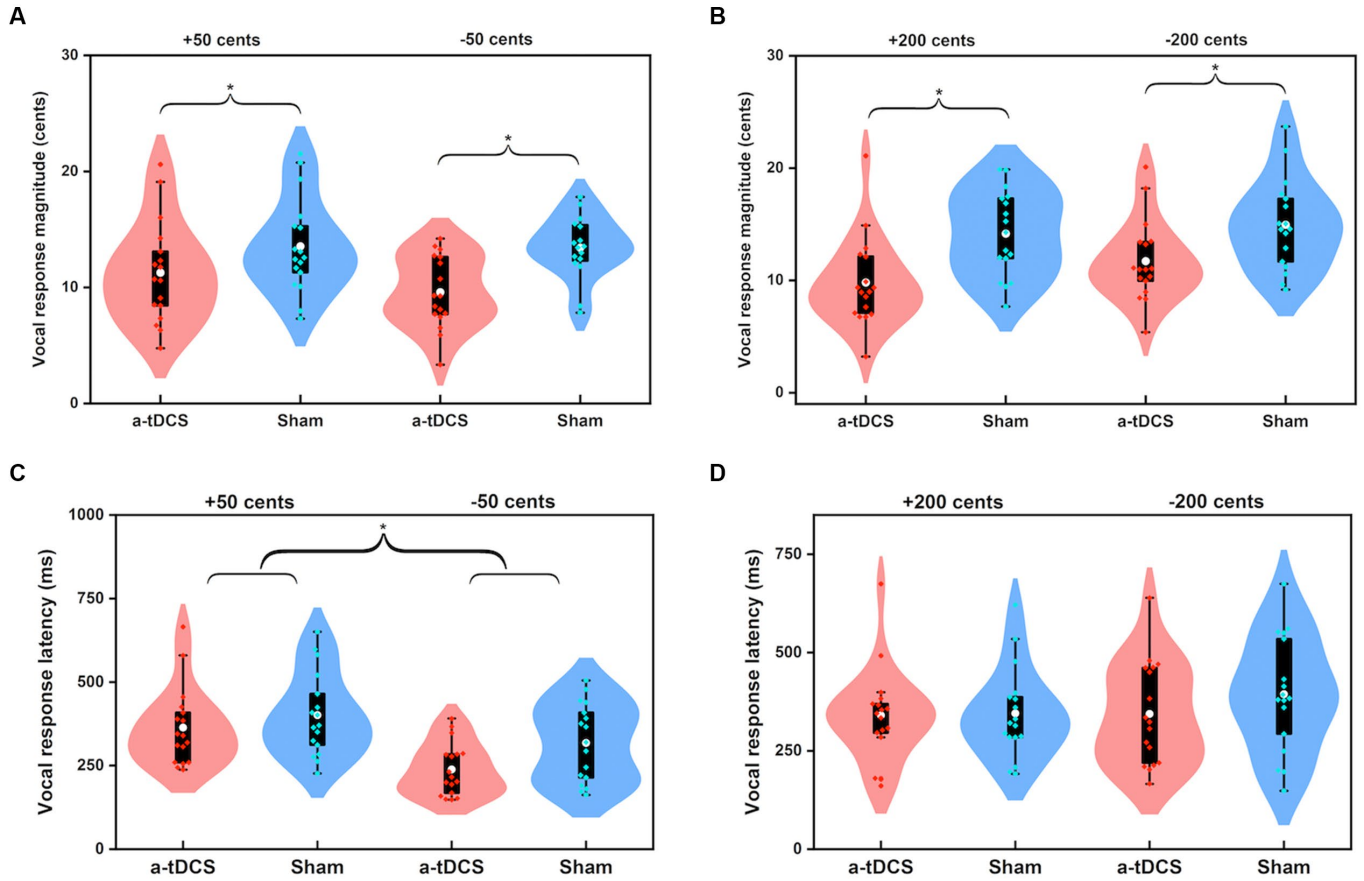
Violin plots illustrating the magnitudes **(A,B)** and latencies **(C,D)** of vocal responses to pitch perturbations of ±50 cents and ±200 cents when active (red) or sham (blue) a-tDCS was applied over the left DLPFC prior to the FAF task. The white dots and box plots represent the medians and ranges from first to third quartiles of the data sets. The red and blue dots represent the individual vocal responses for active and sham a-tDCS over the left DLPFC. The asterisks indicate significant differences across the conditions.

Figure 3 shows the grand-averaged voice *f*_o_ responses to perturbations of ±50 and ±200 cents after active or sham a-tDCS over the left DLPFC. A three-way RM-ANOVA revealed significantly smaller magnitudes of vocal responses elicited by offline a-tDCS over the left DLPFC than sham condition [*F*(1, 16) = 67.972, *p* < 0.001, $\eta_{p}^{2}$ =0.809] (see Figures 4A,B). However, there were no significant main effects of perturbation size [*F*(1, 16) = 0.350, *p* = 0.562] and direction [*F*(1, 19) = 1.051, *p* = 0.321]. The interactions among the three factors were also not significant (*p* > 0.1).

For the peak latencies of vocal responses, upward perturbations elicited significantly longer peak latencies of vocal responses than downward perturbations [F(1, 16) = 5.459, *p* = 0.033, $\eta_{p}^{2}$ =0.254] (see Figures 4C,D). A marginally significant main effect of stimulation condition [*F*(1, 16) = 4.309, *p* = 0.054, $\eta_{p}^{2}$ =0.212] was found, indicating a trend of shorter peak latencies of vocal responses for offline a-tDCS over the left DLPFC than for sham stimulation. The main effect of perturbation size [*F*(1, 19) = 1.627, *p* = 0.220] and interactions among the three factors (*p* > 0.1) were not significant.

### Effects of online vs. offline a-tDCS over left DLPFC

**Figure 5:**
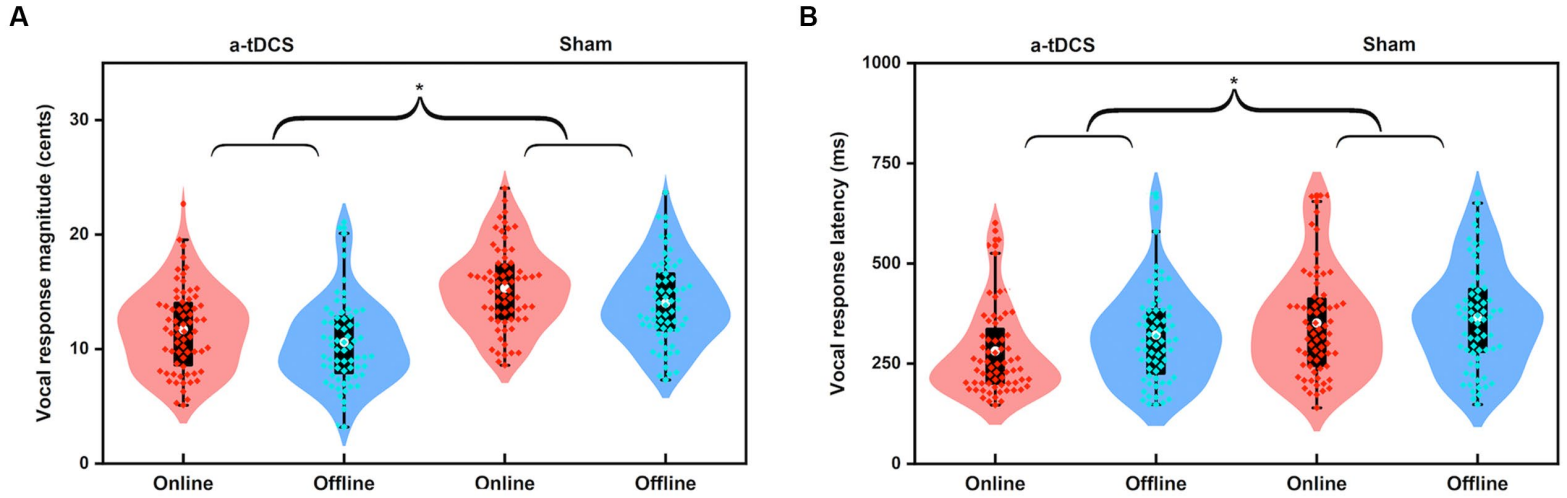
Violin plots illustrating the magnitudes **(A)** and latencies **(B)** of vocal responses to pitch perturbations when active or sham a-tDCS was applied over the left DLPFC prior to (offline) or during (online) the FAF task. The white dots and box plots represent the medians and ranges from first to third quartiles of the data sets. The red and blue dots represent the individual vocal responses for online and offline a-tDCS over the left DLPFC. The asterisks indicate significant differences across the conditions.

A four-way RM-ANOVA conducted on the peak magnitudes of vocal responses revealed a significant main effect of stimulation condition [*F*(1, 16) = 48.477, *p* < 0.001, $\eta_{p}^{2}$ =0.752]. The main effect of stimulation timing [*F*(1, 16) = 3.786, *p* = 0.069] as well as its interaction with stimulation condition [*F*(1, 16) = 0.014, *p* = 0.907] did not reach significance. In addition, the peak magnitudes of vocal responses did not vary as a function of perturbation size [*F*(1, 16) = 0.764, *p* = 0.395] or direction [*F*(1, 19) = 0.001, *p* = 0.972]. The interactions among the four variables were also not significant (*p* > 0.1). These results indicate that, regardless of the timing of a-tDCS and the physical features of pitch perturbations, a-tDCS over the left DLPFC led to significantly smaller vocal compensations for pitch perturbations than sham stimulation (see Figure 5A).

Regarding the peak latencies of vocal responses, there was a significant main effect of stimulation condition [*F*(1, 16) = 16.901, *p* = 0.001, $\eta_{p}^{2}$ = 0.514]. The main effect of stimulation timing [*F*(1, 16) = 3.638, *p* = 0.075] as well as its interaction with stimulation condition [*F*(1, 16) = 0.898, *p* = 0.357] did not reach significance. The interactions among the four variables were also not significant (*p* > 0.2). That is, a-tDCS over the left DLPFC led to significantly shorter peak latencies of vocal compensations for pitch perturbations than sham stimulation, with no significant differences between online and offline stimulations (see Figure 5B). However, across the two stimulation timings, upward and 200 cents perturbations elicited longer peak latencies of vocal responses than downward [*F*(1, 16) = 8.972, *p* = 0.009, $\eta_{p}^{2}$ = 0.359] and 50 cents perturbations [*F*(1, 16) = 31.741, *p* < 0.001, $\eta_{p}^{2}$ =0.665], respectively.

## Discussion

The present study investigated the role of the left DLPFC in vocal feedback control by using a-tDCS during or prior to vocal pitch regulation through auditory feedback. The results showed that both online and offline a-tDCS over the left DLPFC led to smaller peak magnitudes and shorter peak times of vocal compensations for pitch perturbations regardless of their size or direction than sham stimulations. Importantly, there were no significant differences between online and offline stimulations, suggesting that the timing of a-tDCS does not significantly influence its effects on vocal feedback control. In conjunction with previous findings of enhanced vocal compensations for pitch perturbations following cTBS over the left DLPFC (Liu et al., 2020), these findings provide compelling causal evidence supporting the involvement of the left DLPFC in auditory-motor integration for vocal production, corroborating the hypothesis that the left DLPFC exerts top-down inhibitory control over vocal feedback control.

The present study confirms our hyothesis that a-tDCS over the left DLPFC led to reduced vocal compensations for pitch perturbations compared to sham stimulation. In light of the findings that cortical excitablity of the left DLPFC can be increased by a-tDCS (Keeser et al., 2011; Zaehle et al., 2011), this finding implies that enhancing left DLPFC activity with a-tDCS may produce inhibitory modulations of vocal pitch regulation through auditory feedback. Consistently, Liu et al. (2020) found increased vocal compensations for pitch perturbations as a consequence of disrupting activity in the left DLPFC with inhibitory cTBS. These studies collectively establish a causal link between the left DLPFC and vocal feedback control, suggesting that enhancing or inhibiting left DLPFC activity exerts modulatory effects on auditory-vocal integration.

Compensatory vocal adjustment in response to auditory feedback errors has been linked to sensorimotor control of vocal production. Previous studies have shown reduced vocal compensations for pitch perturbations in healthy individuals after speech-sound learning or working memory training (Chen et al., 2015; Guo et al., 2017) and in professional singers following intensive vocal training (Jones and Keough, 2008; Zarate and Zatorre, 2008; Wang et al., 2019). And enhanced vocal compensations for pitch perturbations have been observed in patients with neurological diseases such as AD (Ranasinghe et al., 2017), Parkinson’s disease (PD) (Liu et al., 2012; Chen et al., 2013; Huang et al., 2016; Mollaei et al., 2016), and spinocerebellar ataxia (SCA) (Parrell et al., 2017; Houde et al., 2019; Li et al., 2019). Notably, treatment-induced normalization of this overcompensation behavior has been reported in patients with PD following intensive voice training (Li et al., 2021) or cTBS over the left supplementary motor area (SMA) (Dai et al., 2022), as well as in patients with SCA following cTBS over the right cerebellum (Lin et al., 2022). Therefore, reduced or enhanced compensatory vocal adjustment to perturbed auditory feedback may reflect improved or impaired auditory-motor integration for vocal production. Our results showed that a-tDCS over the left DLPFC led to reduced vocal compensations, suggesting that enhancing left DLPFC activity may facilitate vocal motor control through auditory feedback.

Our finding also showed that a-tDCS over the left DLPFC led to reduced peak times of vocal responses to pitch perturbations. In contrast, prolonged peak times of vocal responses were found when inhibitory cTBS was applied over the left DLPFC (Liu et al., 2020). Other NIBS studies targeting other brain regions have shown that a-tDCS over the right cerebellum prolonged the peak times of vocal responses to pitch perturbations (Peng et al., 2021), while cTBS over the right cerebellum (Lin et al., 2022) or the left or right SMG (Li et al., 2023) shortened them. Prolonged vocal responses to pitch perturbations have been observed in patients with PD (Kiran and Larson, 2001) or aphasia (Johnson et al., 2020), reflecting their impaired sensorimotor integration in speech processing. However, these prolonged vocal responses can be normalized by intensive voice training in patients with PD (Li et al., 2021). Accordingly, our observation of shortened peak times of vocal responses induced by a-tDCS over the left DLPFC leads further support to the idea that enhancing left DLPFC activity facilitates auditory feedback control of vocal production.

The present study found no significant differences between online and offline a-tDCS over the left DLPFC in modulating vocal pitch regulation. This pattern of results is consistent with one recent study that reported comparable effects on vocal pitch regulation of online or offline a-tDCS over the right cerebellum (Peng et al., 2021). These findings suggest that online and offline a-tDCS over the left DLPFC or right cerebellum may have equivalent effects on vocal feedback control. Nevertheless, whether online and offline tDCS have similar or distinct effects on cognitive or motor functions remains open and may be influenced by various factors, including the target site, polarity specificity, and task demands. A meta-analysis study reported similar positive effects of online and offline tDCS over the left DLPFC on cognitive and motor functions (Summers et al., 2016), but other studies found distinct effects of online and offline tDCS over the left DLPFC or the posterior parietal cortex (PPC) on verbal and spatial working memory tasks (Zivanovic et al., 2021), or over the cerebellum on motor learning tasks (Samaei et al., 2017). Therefore, further research is warranted to address the effects of online and offline tDCS over other brain regions on vocal motor control, which would not only elucidate the causality of the underlying neural mechanisms but also optimize the parameters and protocols of tDCS for potential therapeutic applications in motor speech disorders.

Our results, along with the findings of Liu et al. (2020), demonstrate that modulating left DLPFC activity affects vocal pitch regulation in a bidirectional manner: enhancing or inhibiting its activity decreased and increased vocal compensations for pitch perturbations, respectively. These findings can be accounted for a top-down inhibitory mechanism mediated by the left DLPFC (Liu et al., 2020), which relies on two key aspects: (1) the left DLPFC mediates cognitive functions such as attentional control, working memory, and inhibitory control, which have been demonstrated to be essentially involved in vocal feedback control (Tumber et al., 2014; Liu et al., 2015; Guo et al., 2017; Ranasinghe et al., 2017); (2) the DLPFC has reciprocal connections to auditory and motor regions (Selemon and Goldman-Rakic, 1988; Romanski et al., 1999). This mechanism suggests that the left DLPFC may exert top-down inhibitory control over the interaction between auditory and motor representations of speech sounds that prevents excessive compensatory vocal adjustment for feedback perturbations to ensure precise and stable speech production (Houde and Nagarajan, 2011). Similarly, Ranasinghe et al. (2017) proposed that the prefrontal cortex may generate an inhibiotry influence on vocal motor control that leads to incomplete compensations for feedback errors. Therefore, dysfucntions of this top-down inhibitory process would result in enhanced vocal compensations for feedback errors, as evidenced by patients with AD who showed enhanced vocal compensations and reduced left DLPFC activity (Ranasinghe et al., 2017, 2019). Conversely, improvement of this top–down inhibitory control process would result in reduced vocal compensation for feedback errors, as evidenced by patients with PD who showed improved vocal loudness following intensive voice training that was correlated with reduced vocal compensations (Li et al., 2021) and increased activity in the DLPFC (Liotti et al., 2003; Narayana et al., 2010). This mechanism also helps explain why working memory training led to reduced vocal compensations for pitch perturbations (Guo et al., 2017). However, the precise neural mechanisms and pathways by which the left DLPFC exerts top-down control over vocal feedback control are largely unknown and warrant further investigation.

Several limitations of the present study should be acknowledged. First, the present study only used a single session of a-tDCS over the left DLPFC to evaluate their immediate effects on vocal pitch regulation. This design leaves open the long-term effects as well as the optimal parameters of a-tDCS for enhancing vocal feedback control. Second, the present study did not collect neuroimaging data such as ERP and fMRI concurrently with the acoustic data, limiting our ability to elucidate the neural mechanisms underlying the causal role of the left DLPFC in vocal feedback control. Finally, the conventional tDCS protocol employed in the present study may induce widespread currents to other brain regions due to the limited focality of stimulation (de Berker et al., 2013). In subsequent studies, high-definition tDCS (HD-tDCS) that offers improved focality of brain stimulation should be considered to verify the present findings.

## Conclusion

In summary, the present study showed that a-tDCS over the left DLPFC led to reduced peak magnitudes and prolonged peak times of vocal compensations for pitch perturbations relative to sham stimulation, regardless the size or direction of the pitch perturbation and the timing of the stimulation. These findings provide causal evidence that a-tDCS over the left DLPFC can facilitate auditory-motor integration for rapid and precise control of vocal production. The present study, together with Liu et al. (2020), lends support to the hypothesis of a top-down mechanism mediated by the left DLPFC that exerts inhibitory influences on vocal feedback control.

## Data availability statement

The raw data supporting the conclusions of this article will be made available by the authors, without undue reservation.

## Ethics statement

The studies involving human participants were reviewed and approved by Institutional Review Board of The First Affiliated Hospital of Sun Yat-sen University. The patients/participants provided their written informed consent to participate in this study.

## Author contributions

YC, DP, and HL contributed to the design of the study. YC, DP, YZ, XC, XW, and JL contributed to the acquisition and analysis of data. YC, XW, PL, and HL contributed to drafting the manuscript and preparing the figures. All authors have reviewed and approved the contents of the paper.

## Funding

This study was funded by grants from the National Natural Science Foundation of China (Nos. 82172528, 81772439, 81972147, 82102660, 82102648), Guangdong Basic and Applied Basic Research Foundation (No. 2022A1515011203, 2023A1515011758), Guangdong Province Science and Technology Planning Project (No. 2017A050501014), and Guangzhou Science and Technology Programme (No. 201604020115).

## Conflict of interest

The authors declare that the research was conducted in the absence of any commercial or financial relationships that could be construed as a potential conflict of interest.

## Publisher’s note

All claims expressed in this article are solely those of the authors and do not necessarily represent those of their affiliated organizations, or those of the publisher, the editors and the reviewers. Any product that may be evaluated in this article, or claim that may be made by its manufacturer, is not guaranteed or endorsed by the publisher.
